# Machine learning and deep learning models for predicting colorectal cancer metastases: A comprehensive review

**DOI:** 10.1016/j.ejro.2026.100747

**Published:** 2026-04-16

**Authors:** Mikiyas Amare Getu, Tesfaye Amare, Kefeng Li, Anam Mehmood, Yonas Fissha Adem, Pablo Santos, Hu Yan

**Affiliations:** a Fudan University, School of Nursing, Shanghai, China; b School of Nursing, Woldia University, Ethiopia; c Global and Planetary Health Working Group, Institute of Medical Epidemiology, Biostatistics and Informatics, Martin Luther University Halle-Wittenberg, Halle (Saale), Germany; d Faculty of Technology, Woldia University,Ethiopia; e Faculty of Applied Sciences, Macao Polytechnic University, Macao Special Administrative Region of China; f School of Psychology, Sichuan Normal University, Chendgu, China; g Department of Public Health, Dessie College of Health Sciences, Dessie, Ethiopia

**Keywords:** Cancer, Metastasis, Artificial intelligence, Deep learning, Machine learning

## Abstract

Colorectal cancer (CRC) is a leading cause of cancer-related mortality worldwide, largely due to the development of distant metastases, which is associated with poor survival outcomes. Early detection and accurate prediction of colorectal cancer metastasis can significantly improve patient outcomes. Conventional diagnostic approaches, including imaging and biomarkers, are often limited by suboptimal sensitivity and inter-observer variability. In recent years, machine learning (ML) and deep learning (DL) models have emerged as powerful tools capable of analyzing complex, high-dimensional clinical, imaging, and molecular data to enhance metastasis prediction. This review provides a comprehensive overview of ML and DL approaches for the early prediction of CRC metastasis, highlighting gaps in comparative studies. We explore DL techniques, including convolutional neural networks (CNNs), and alternative approaches, along with their architectures and layer types. The most commonly used CNN models such as GoogleNet, VGGNet, ResNet, and U-Net have demonstrated effectiveness in identifying complex patterns of data. These predictive models have improved individualized treatment strategies, leading to enhanced patient outcomes owing to the integration of multi-modal data including imaging, clinical data, histological data and employing transfer learning. This review also examines the applications of ML and DL in predicting CRC metastasis to specific sites such as lymph nodes, liver, lungs, bones, and peritoneum. While traditional ML algorithms, including logistic regression and random forests remain valuable, DL models incorporating radiomics and transfer learning, often achieve superior performance. Finally, we explore how the computational costs and resource implications of ML and DL technologies need attention in clinical contexts. Challenges such as the availability of high-quality datasets, interpretability, and ethical concerns are examined, and appropriate solutions are discussed. Future research should focus on developing explainable ML/DL models, optimizing computational resources, establishing ethical frameworks, validation of model performance across diverse populations, along with the incorporation of molecular and genomic data.

## 1 Introduction

Colorectal cancer (CRC) is a significant global health problem, ranking third in incidence and second in mortality, with approximately 1.9 million new cases and 0.9 million deaths worldwide in 2022 [1]. The global burden of CRC is expected to rise to 3.2 million cases by 2040 [2]. The majority of CRC cases and deaths are related to modifiable factors such as high-level alcohol consumption, smoking, physical inactivity and excess body weight. In addition, a large proportion of CRC incidence and mortality is preventable through regular monitoring, follow-up and treatment [3].

Among new CRC cases, approximately 20% present with metastatic disease at diagnosis, while nearly 50% of early-stage CRC patients will eventually develop distant metastases during the course of their disease [4]. Colorectal cancer metastasis involves a complex pathophysiological process where cancer cells spread from the primary tumor to distant organs, most commonly the liver, lungs, lymph nodes, and peritoneum [5]. Liver metastasis, occurs in about 50% of metastatic CRC cases, likely due to the anatomical proximity to colorectum [6]. Despite improvements in surgical techniques, systemic therapy, and targeted treatments, outcomes for metastatic CRC remain poor. The 5-year survival rate is approximately 14% [5]. For synchronous and metachronous liver metastases, the 5-year survival rates were reported as 3.3% and 6.1%, respectively [7].

Early detection and accurate prediction of metastasis can significantly improve patient outcomes by enabling personalized treatment strategies, leading to improved survival rates and improved quality of life [8]. Predictive information can guide individualized surveillance strategies, inform the use of neoadjuvant and adjuvant therapies, and support timely surgical or systemic interventions. This targeted approach not only improves patient care but also contributes to more efficient resource allocation within healthcare systems. However, traditional methods, such as radiological assessment, histopathology, and serum biomarkers-are often limited by suboptimal sensitivity and specificity, variability between observers and difficulty capturing subtle or high-dimensional patterns associated with metastatic potential [9]. In contrast, machine learning (ML) and deep learning (DL) approaches offer advantages in analyzing complex datasets and identifying patterns that may be missed by conventional methods. These relatively new technologies can provide more precise metastasis predictions, facilitating earlier and more accurate intervention, thereby contributing to better patient outcomes [10].

To address these limitations, data-driven computational approaches have recently been used for CRC metastasis prediction. Machine learning (ML) models using structured clinical, pathological, and imaging-derived data have shown promising performance across several metastatic sites. Large population-based studies have also shown that ML models trained on clinicopathological variables can predict lung metastasis, with balanced random forest approaches successfully applied to multi-center datasets [11]. Similarly, ML models such as XGBoost have outperformed experienced clinicians in the preoperative prediction of lymph node metastasis (LMN) using ultrasound-derived and laboratory features [12].

Concurrently, deep learning (DL) methods based primarily on convolutional neural networks—have been explored more intensively for CRC metastasis prediction, especially in imaging-intensive applications. CRC most frequently metastasizes to lymph nodes and the liver, while metastases to the lung, bone, and peritoneum occurs less often [13], [14]. DL architectures such as ResNet, VGG, and U-Net have demonstrated strong capability in learning complex spatial patterns from medical images.

Moreover, DL models applied to radiological imaging appear to have potential predicting metachronous liver metastasis from the fact that abdominal CT images features extracted through pre-trained VGG-16 network, along with clinical variables, outperformed models based on clinical data alone in predicting 5-year liver metastasis [15]. Despite the growing number of studies on the application of ML and DL, the literature remains highly fragmented with much heterogeneity in terms of data source, model architecture, evaluation metrics, and clinical endpoints [16]. Consequently, existing reviews usually focus either on cancer prediction in general or on isolated AI methods without providing a synthesis that is disease-specific and biased toward the metastatic avenue. Furthermore, clinical interpretability, external validation, and real-world application of these models have also received the least attention [17]. Therefore, this review will fill these gaps through a comprehensive review and comparison of existing approaches in ML and DL with an emphasis on prediction of CRC metastasis, detailing the methodological strengths, weaknesses, and potential for translating findings into practice.

The purpose of this review is to: (a) comprehensively evaluate and summarize the current advancements in deep learning and machine learning models for the prediction of colorectal cancer metastasis; (b) review various methodologies, algorithms, and techniques employed in these predictive models, and assess their performance using relevant indicators; (c) explore the potential clinical applications and implications of these models, providing insights to enhance early prediction of metastasis, improve patient outcomes, and inform treatment strategies for colorectal cancer metastasis; (d) identify challenges and future research directions.

### 1.1 Contributions and novelty of the review

This review makes several novel contributions to the existing literature. First, this review provides a comprehensive synthesis of ML and DL algorithms developed for the purpose of predicting colorectal cancer metastasis with a focus on clinically meaningful metastatic sites including lymph nodes, liver, lung, bone, and peritoneum. Unlike other reviews that address colorectal cancer prediction in general terms, this study offers a site-specific comparative view.

Second, it systematically compares traditional ML and advanced DL methods, across different data modalities, performance metrics, datasets, and evaluation strategies. Third, this study critically analyzes the recent rise of radiomics and integrated with multimodal learning frameworks that incorporate imaging, clinical, and pathological information, including the added value these frameworks contribute as well as their limitations.

Finally, the study identifies key methodological inconsistencies, discusses translational challenges, and proposes future research directions to improve the clinical relevance and reproducibility of AI-based models for the prediction of metastasis.

## 2 Review methodology

We conducted a comprehensive search on the following databases: Web of Science (https://www.webofscience.com), Google Scholar (https://scholar.google.com), Scopus (https://www.scopus.com), ScienceDirect (https://www.sciencedirect.com), IEEE Xplore (https://ieeexplore.ieee.org), Springer (https://link.springer.com), and PubMed (https://pubmed.ncbi.nlm.nih.gov). Together, these databases provide extensive coverage across wide range research disciplines. The search focused on titles and abstracts, with the reference period extending from the inception of each database to August 2024.

The search strategy employed specific keywords and Boolean operators to ensure the inclusion of pertinent literature. The complete search strategy was: (“deep learning” OR “machine learning” OR “artificial intelligence”) AND (“colorectal cancer” OR “colon cancer” OR “rectal cancer”) AND (“metastasis” OR “metastases”) AND (“prediction”).

Our literature search identified 280 studies deemed relevant to this review. After removing duplicates and excluding studies that did not meet the inclusion criteria, we included 38 English-language articles in the final review. The inclusion criteria specified articles that addressed the use of deep learning and machine learning for predicting colorectal cancer metastasis, regardless of the metastatic site involved. We excluded studies focused on colorectal cancer metastasis diagnosis, evaluation of treatment response, survival analysis, as well as case reports, editorials, and reviews.

Data extraction from the selected papers was conducted by JBP, AP, and LFSP. The data extraction table (Table 1) included the following information: author, year; study design; purpose of the study; site of the primary tumor; imaging modality; type of treatment; deep learning or machine learning model; feature extraction; data augmentation; results based on performance metrics; and the name of the dataset or data source.

**Table 1:** General summary and results of studies using machine learning models for prediction of colorectal cancer metastasis.

Author (year)	Study purpose	Tumor site	Treatment	Imaging modality	Machine learning model	Number of samples	Results
Zhang et al. (2023) [11]	To predict lung metastasis	CRC	No neoadjuvant therapy	Clinicopathological factors	KNN, SVM, DT, RF, and BRF	46,037 patients from SEER database and 2779 patients from a multi-center external validation set.	The BRF model performed best with AUC of 0.874 and an average precision (AP) of 0.184.
Kudo et al. [29]	To predict risk of lymph node metastasis	CRC	Underwent endoscopic resection plus surgical resection	Demographic and clinical data	ANN	Training cohort (n = 3134) External validation (n = 939)	AUC: 0.84
Takamatsu et al. [9]	To predict lymph node metastasis	CRC	Underwent endoscopic resection plus surgical resection	Histopathology	RFC	Training cohort (n = 277) Test cohort (n = 120)	The sensitivity and specificity were 80.0% and 94.5% respectively. Average AUC: 0.822
Li et al. [33]	To predict liver metastasis	CRC	Postoperative	Free-text medical record data and laboratory data (Fusion model)	XGBoost, SVM, KNN, DT, RF, and NLP models such as BERT.	Training cohort (n = 239), Internal validation cohort (n = 60), External validation cohort (n = 52)	Fusion models accuracy: 80.8%, precision: 80.3%, recall: 80.5%, and F1 score: 80.8%
Huang et al. (2023) [12]	To predict lymph node metastasis	Rectal cancer	Underwent total mesorectal excision	3D Endorectal ultrasound and clinical and laboratory data	SVM, XGBoost, RF and LightGBM	Training set (n = 88) Test set (n = 38)	XGBoost model had better performance (Accuracy:84.21%, sensitivity:75%, specificity: 90.9%, and AUC: 0.82) than radiologist
He et al. [30]	To predict regional lymph node metastasis	CRC	CRC radical surgery	18F-FDG PET/CT scans	LR, SVM, RF, and XGBoost	Training cohort (n = 144) Test set (n = 55)	Logistic regression and XGBoost model performed best (AUC = 0.866 and AUC = 0.903 respectively)
Jin et al. [13]	To predict bone metastasis	CRC	-	Pelvic MRI	ANN, RF, DT and SVM	Training cohort (n = 429) Validation cohort (n = 185)	RFM has the best predictive efficacy. Training set AUC: 0.926 Internal validation set AUC: 0.919.
Li et al. [34]	To predict liver metastasis	CRC	--	Preoperative CT	LR, XGBoost, KNN, SVM, and RF	48 patients (24 patients with LM and 24 patients without LM)	RF model showed the best performance achieving an accuracy of 96.0%, sensitivity of 99.7%, specificity of 92.9%, and an AUC of 0.991
Yongfei et al. (2024) [35]	To predict lymph node metastasis	Rectal cancer	Preoperative	Multiparametric MRI	Radscore, clinical, and combined models	318 patients	The AUC of the combined model was higher than other models in the training (0.837) and the external validation cohort (0.880)
Qiu et al. [36]	To predict risk of liver metastasis	Rectal cancer	Not preoperative neoadjuvant therapy	Clinicopathological data	RF, LGBM, XGB, MLP, LR, and KNN	SEER database: Training cohort (n = 15966) Testing cohort (n = 3992) External validation cohort (n = 924) from two hospitals in China	The XGB model showed the best predictive power, with AUC: 0.926, accuracy: 0.919, sensitivity: 0.740, and specificity: 0.765.

## 3 Radiomics and multimodal learning in colorectal cancer metastasis

Radiomics is the extraction of quantitative features from medical images in high-throughout, capturing tumor shape, texture, and intensity patterns that cannot be perceived by the human eye. It has played a critical role in a multimodal AI framework for predicting colorectal cancer metastases, mainly for the improvement of risk stratification via combination with clinical and pathological data [18].

According to recent studies, radiomics-based models especially those combined with deep learning architecture have overtaken single-modality approaches in predicting liver and LMN. [19]. However, challenges remain regarding feature reproducibility, segmentation variability, and the lack of standardized pipelines [20].

## 4 Machine learning

ML methods focus on developing algorithms that enable computers to learn from and make predictions or decisions based on data. Learning from experience is the basis of human and animal learning. Therefore, most adaptive or machine learning work emphasizes enabling computers to learn through experience. The primary aim of ML is to enable computers automatically identify patterns and make inferences from large volumes of data. In other words, ML automates statistical inferences methods so that patterns which are not immediately apparent to human analysts can be identified. ML algorithms have been developed for applications such as speech, character recognition, image recognition, and control systems [21].

In the context of predicting colorectal cancer metastasis, an image classification task would involve training an algorithm on a dataset that contains a large number of images of both metastatic and non-metastatic CRC. The algorithm would thus “learn” to distinguish between these two classes based on the training data [22].

There are three types of ML: supervised learning, unsupervised learning, and reinforcement learning.

### 4.1 Supervised learning

Supervised learning involves training algorithms on labeled data, in which the input-output pairs are known. This enables the system to learn and predict outcomes for new, yet “unseen” data. This type of learning can be applied to both classification and regression tasks. Common algorithms include decision trees and support vector machines. In practice, supervised learning requires a training dataset with input features and corresponding target values, allowing the model to learn the relationship between them [23].

### 4.2 Unsupervised learning

Unsupervised learning involves training on unlabeled data with the aim of identifying hidden patterns or groupings within the data based on assumed structural properties. Techniques include clustering methods such as k-means and dimensionality reduction methods. Dimensionality reduction techniques transform data from high-dimensional space to lower-dimensional space and include principal component analysis, manifold learning, factor analysis, random projections and autoencoders. A prominent example of unsupervised learning is clustering. In both clustering and dimensionality reduction techniques, computational complexity is a significant consideration, given the goal of using large datasets without relying on supervised labels [24].

### 4.3 Reinforcement learning

Reinforcement learning occupies an intermediate position between supervised and unsupervised learning. In reinforcement learning, the data typically provide only feedback on whether an action is correct or not. If an action is incorrect, the challenge is to determine the correct action. Reinforcement learning algorithms often draw on concepts from control theory, such as policy iteration, value iteration, rollouts, and variance reduction, while also introducing innovations to address specific challenges in machine learning, such as large-scale problems, minimal assumptions about unknown dynamic environments, and the use of supervised learning architectures to represent policies. There is a strong connection between reinforcement learning and learning in psychology and neuroscience. The best example is the application of reinforcement learning algorithms to predict a monkey's response when associating a light stimulus with a sugar reward [25].

Although these three machine learning paradigms—supervised, unsupervised, and reinforcement learning—help to organize ideas, much current research involves combinations of these categories. For instance, semi-supervised learning leverages unlabeled data to augment labeled data within a supervised learning framework, and discriminative training merges architectures developed for unsupervised learning with optimization techniques that utilize labels. Various factors influence the design of learning algorithms across all these paradigms, including whether data are available in batches or arrive sequentially over time, the method of data sampling, the need for learned models to be interpretable by users, and robustness issues that arise when data do not conform to prior modeling assumptions [26].

### 4.4 Machine learning models application in the prediction of colorectal cancer metastasis

This section reviews the application of machine learning models for predicting colorectal cancer metastasis. Across the studies summarized in Table 1, classical machine learning models depicted strong predictive performance for colorectal cancer metastasis, particularly when large clinicopathological datasets were available. The machine learning models most commonly utilized for predicting lymph-node, liver, lung, and bone metastasis among CRC patients include logistic regression (LR), support vector machines (SVM), random forest classifiers (RFC), balanced random forests (BRF), artificial neural networks (ANN), extreme gradient boosting (XGBoost), k-nearest neighbors (KNN), bidirectional encoder representations from transformers (BERT), and Light Gradient Boosting Machine (LightGBM) [27].

In supervised machine learning for metastasis prediction, each patient is represented by a feature vector [28].

$$x_{i}\in R^{d},$$

with a corresponding binary label

$$y_{i}\in \{0,1\},$$

where $y_{i}=1$ denotes the presence of metastasis. Models aim to learn a function

$$f(x_{i};\theta )\to {\hat{y}}_{i}$$

by minimizing a loss function $L(y_{i},{\hat{y}}_{i})$over the training dataset.

Takamatsu et al. (2019) [9] conducted a retrospective study on 397 T1 CRC patients to predict LMN using RFC based on morphologic parameters from whole H&E slide images and cytokeratin immunohistochemistry. The sensitivity and specificity of the machine learning model in predicting LNM were 80.0% and 94.5%, respectively, indicating that ML can be a superior predictor compared to conventional methods and can aid in determining treatment strategies for T1 CRC patients.

Kudo et al. (2021) [29] developed an ANN to identify 3134 T1 CRC patients at risk of LMN. The model used data on patients’ age, sex, tumor size, tumor site, morphology, lymphatic and vascular invasion, and histologic grade. The ANN model, with an AUC of 0.83, outperformed existing guidelines, in which depth of invasion and tumor budding were used as predictors of LNM (AUC of 0.73).

He et al. (2021) [30] developed a regional LMN prediction model for 199 CRC patients based on radiomic features of extracted from PET/CT scans. The logistic regression (AUC 0.866) and XGBoost (AUC 0.903) models performed best among the evaluated machine learning models.

Zhang et al. (2023)[11] developed and validated an AI prediction model for lung metastasis in colorectal cancer patients. This model was based on clinicopathological characteristics from 46,037 CRC patients in the Surveillance, Epidemiology, and End Results (SEER) database and 2779 CRC patients from a multi-center hospital. Six machine learning models — LR, SVM, DT, RFC, KNN, and BRF—were utilized. Among these models, the BRF model demonstrated the best performance, with an Area Under the Receiver Operating Characteristic Curve (AUC) of 0.874 and an average precision (AP) of 0.184. This study highlights the advantage of ensemble methods in handling class imbalance common in metastatic datasets.

Huang et al. (2023) [12] investigated the preoperative prediction of LMN in rectal cancer patients using three-dimensional endorectal ultrasound and clinical laboratory data. The XGBoost model exhibited the best performance, with an AUC of 0.82, compared with experienced radiologists (whose AUC was 0.60).

Liu et al. [31]showed in the population-based studies using SEER data that the gradient boosting framework was able to consistently achieve higher results than logistic regression on liver metastasis prediction, highlighting the significance of nonlinear feature interactions on metastatic progression.

Jin et al. [32] developed a machine-learning–based early warning system using gray-level co-occurrence matrix (GLCM) texture features extracted from MRI to detect pelvic bone metastases in patients with colorectal cancer. By comparing multiple machine learning models, they found that the random forest model achieved the best performance, demonstrating high diagnostic accuracy with an AUC of approximately 0.92 in both training and validation cohorts, indicating its potential benefit for early and noninvasive detection of bone metastases.

Finally, Li et al. [33] developed a fusion model integrating free-text medical record data and structured laboratory data to predict liver metastasis among 1463 postoperative CRC patients. The fusion model employed machine learning techniques, including XGBoost, SVM, KNN, decision tree (DT), random forests (RF), extra trees and NLP models such as BERT. The fusion model demonstrated superior predictive performance, with an accuracy of 80.8%, a precision of 80.3%, a recall of 80.5%, and an F1 score of 80.8% for predicting liver metastasis.

## 5 Deep learning

DL is one of the most commonly used AI tools today in the field of medicine [37]. Its algorithms use hidden layers to manage complex clinical and imaging data with diverse structure [38]. DL consist of neural network that automatically learn hierarchical features through convolutional layer specialized for processing spatial data and back propagation [39]. Backpropagation is a learning algorithm that updates the network's parameters—weights and biases—by calculating the gradient of the loss function and propagating errors backward through the networks by minimizing a loss function ℒ (y, ŷ). Neural networks, which form the foundation of deep learning models, are comprised of neurons with activation functions and parameters, including weights and biases [40]. For a network with parameters θ, gradients are computed using the chain rule: ∂ℒ / ∂θ

Parameters are updated iteratively using gradient descent: θ^(t + 1) = θ^(t) − η · (∂ℒ / ∂θ)

where η denotes the learning rate [41].

As with ML, also DL architectures are categorized into supervised, unsupervised and hybrid learning methods. In supervised learning, models learn from labeled datasets. These labels may represent class categories in classification tasks or continuous values in regression tasks. The objective is to optimize model parameters to minimize a defined loss function, ensuring accurate predictions. Conversely, unsupervised learning function without labeled data and, focusing on uncovering inherent patterns or structures within datasets. Techniques such as principal component analysis and clustering are examples, where the emphasis is on exploring data relationships and discovering latent features without explicit labels. Hybrid deep networks refer to architectures that combining various types of deep learning approaches to achieve improved results [40]. Deep learning architectures includes convolutional neural network, recurrent neural networks (RNNs), autoencoders (AEs), restricted Boltzmann machines (RBMs), and deep belief networks (DBNs) [38]. Among these, CNN, autoencoders, and deep belief neural networks have proven particularly successful in colon cancer research [38]. Deep learning can be used to develop biomarkers for prediction patient outcomes directly from conventional histological images [42].

The following sections will review different types of deep learning techniques, architectures, layers and models used for prediction of CRC metastasis.

### 5.1 Convolutional neural network (CNN)

The CNN is a widely used deep learning architecture inspired by the natural visual perception mechanisms found in the visual cortex of animals, which are responsible for interpreting signals sent by retinal neurons corresponding to light and shapes [43]. A CNN is a specialized type of neural network that uses convolutional layers, which are fundamental for feature extraction from input data, usually images. Unlike traditional neural networks that use general matrix operations, CNNs have emerged as a dominant architecture in medical image processing and analysis, largely due to their ability to effectively process and extract useful features from visual data (images) [40].

A CNN consists of three main types of layers: —input layer, hidden, and output layer [44]. The hidden layers of a CNN model is composed of convolutional, nonlinear, pooling, dropout and fully connected layers. When stacked in deep learning architectures alongside activation layers, these layers enable the neural network to be sensitive to minute details and insensitive to large irrelevant details [39], [40]*.*

### 5.2 CNN layers

#### 5.2.1 Convolutional layer

The convolutional layer consists of a set of filters or kernels that move across the input image. At each position, the filter multiplies its values with the corresponding pixel values in the input image (element wise) and then adds them together to produce a single value. This process is called a dot product. These filters extract high-level features, such as horizontal or vertical edges, patterns, and textures [44]. The result of this process is a feature map, which contains data reflecting the features detected by each filter [38].

Mathematically, given an input feature map

X ∈ ℝ^(H×W) and a convolution kernel

K ∈ ℝ ^ (k × k), the convolution operation is defined as:

Y (i, j) = (X * K) (I, j) = Σ _m Σ _n X (I + m, j + n) · K (m, n)

where (i, j) denotes the spatial location in the output feature map Y.

Stride s controls the step size of the kernel movement, and padding p is applied to preserve spatial dimensions [45].

#### 5.2.2 Non‑linearity layer

The performance of CNNs is affected by the implementation of non-linear activation functions in the non-linear layer, which increases the non-linearity of the network. Some popular types of nonlinear activation functions include the Rectified Linear Unit (ReLU), sigmoid and tangent function (Tanh) [46]. The sigmoid function is mostly used for models that predict probabilities, as it produces outputs between 0 and 1. The Tanh function ranges between −1 and 1 and is symmetric about the origin unlike sigmoid function. In addition, ReLU is the most commonly used activation function in CNNs because it can compute faster than others and also helps overcome the vanishing gradient problem.

Common activation functions used in CNNs are defined as follows:

Rectified Linear Unit (ReLU): f(x) = max (0, x)

Sigmoid: f(x) = 1 / (1 + e^(−x))

Hyperbolic tangent (Tanh): f(x) = tanh(x)

These functions introduce non-linearity into the network, enabling the learning of complex decision boundaries [47].

#### 5.2.3 Pooling layer

A pooling layer is used to reduce the dimensionality of feature maps, thereby decreasing the computational load and controlling over fitting. Pooling operation can be performed in three ways: maximum pooling, average pooling, and minimum pooling. The most common type of pooling is maximum pooling, which selects the maximum value from each patch of the feature map, effectively down sampling the input representation. Minimum pooling selects the smallest value, while average pooling calculates the average value of the pixels in the feature map [38], [44].

For max pooling with a pooling window Ω_ (i, j), the pooling operation is defined as:

Y (i, j) = max_ {(m, n) ∈ Ω_ (i, j)} X (m, n)

This operation reduces spatial dimensionality while preserving the most salient features.

#### 5.2.4 Dropout layer

A dropout layer combats overfitting by randomly deactivating a fraction of neurons during training, forcing the network to learn more robust and generalized features for improved performance on unseen data [46].

#### 5.2.5 Fully connected layer

This layer serves as a final stage that takes the high-level, abstracted features produced by the convolutional and pooling layers and uses them to classify the input data into various categories. The feature maps are flattened into one-dimensional vector [39] [48].

### 5.3 CNN models

Convolutional Neural Network (CNN) models are highly effective for medical image classification, detection, and segmentation. Typical models such as AlexNet, GoogleNet, VGG, and ResNet are primarily used for image classification [49]. For object detection, models like R-CNN, Faster R-CNN, YOLO, PFN, PSPNet, and EfficientNet are commonly used. Regarding semantic segmentation, which associates each pixel of an image with category or object, the most popular models include Fully Convolutional Networks (FCN), PSPNet, DeepLab, U-Net, and SegNet [44], [50]*.* For instance, segmentation tasks, Mask R-CNN and YOLACT are widely utilized. All these models use the convolutional components of fundamental CNN architectures—such as AlexNet, GoogLeNet, VGG, ResNet, Inception, MobileNet, DenseNet, and EfficientNet—as feature extractors, replacing fully connected layers with other techniques suited to specific tasks [44], [51], [52]*.* Among all of these architectures, some of them are used for colorectal cancer metastasis prediction such as VGGNet [15] ResNet [14], and U-Net [53] for liver, peritoneum and LMN, respectively.

#### 5.3.1 AlexNet model

AlexNet, the pioneering convolutional neural network (CNN) architecture, was proposed by Alex Krizhevsky, Geoffrey Hinton, and Ilya Sutskever. It became the first model to win the ImageNet Large Scale Visual Recognition Challenge (ILSVRC) in 2012, achieving a top-5 test accuracy of 84.6% on the ImageNet dataset, which contains more than 15 million images [54]. The model was trained on two GTX 580 GPUs for 5–6 days [52].

#### 5.3.2 GoogLeNet model

GoogLeNet, is the state-of-the-art performance on ImageNet, proposed by Christian Szegedy of Google with the objective of reducing computational complexity as compared to other traditional CNN architectures [55], [52].

GoogleNet improved the network into Inception v2 and Inception v3 aimed at improving accuracy and efficiency. These advancements included integrating techniques such as batch normalization, dimensionality reduction and convolution kernel factorization to reduce complexity. These innovations together contributed to higher accuracy while making the networks more computationally efficient [46], [56].

#### 5.3.3 Visual geometry group network (VGGNet)

VGGNet is a CNN architecture introduced by the visual geometry group at the University of Oxford, known for its simplicity and effectiveness in image classification tasks. It achieved first and second places in object detection and classification tasks with 7.3% error rate in the ILSVRC competition in 2014 [57].

#### 5.3.4 Residual network (ResNet)

ResNet, developed by Kaiming He [58] was designed to overcome the vanishing gradient problem, which affected many earlier DL models. The vanishing gradient problem occurs during the training of deep neural networks when the gradients of the loss function become very small. ResNet is a type of deep learning architecture that is particularly suitable for image recognition and object detection [58], [52].

ResNet models vary in depth, including ResNet34, ResNet50, ResNet101, ResNet152, and even ResNet1202. ResNet50, the most popular variant, contain 49 convolutional layers and one fully connected layer at the end of the network [46], [59]. Among these architectures, ResNet, VGG, and Inception are frequently used for image classification and colon cancer analysis [38].

#### 5.3.5 Dense convolutional network (DenseNet)

DenseNet was developed by Gao Huang et al. [60], connects each layer to every other layer in a feed-forward fashion, alleviating the vanishing gradient problem, promoting feature reuse, and substantially reducing network parameters. DenseNet consists of dense blocks and transition blocks, which are placed between two adjacent dense blocks. DenseNet introduces direct connections of any layer to subsequent layers to ensure efficient information flow in the layers. Its ability to capture complex spatial and hierarchical features makes DenseNet particularly relevant for predicting metastasis in colorectal cancer patients [60].

### 5.4 Alternative deep learning architectures

This section reviews alternative deep learning architectures for modeling and understanding complex data patterns, expanding beyond traditional neural networks like CNN. Alternative deep learning architectures include recurrent neural networks (RNNs), autoencoders, restricted Boltzmann machines (RBMs), and deep belief networks (DBNs). Traditional neural network approaches are limited in their ability to understand and process sequential or variable-length data because they can handle only a fixed- size input vector (e.g., an image or a video frame) and produce a fixed-size output vector (e.g., probabilities of different classes). In addition, traditional approach operates within a fixed number of computational steps (e.g. the number of layers in the model) [46].

### 5.5 Deep learning models application in prediction of colorectal cancer metastasis

This section reviews the current applications of deep learning models in predicting colorectal cancer metastasis. CRC is commonly metastasizes to the lymph nodes [61], [62], [63]and the liver [64] whereas it is less frequently metastasized to the lung [65], bone [13], and peritoneum [14]. Various DL models such as ResNet-50 [61], ResNet152 [62], VGG-16 [15], VGG-19 [66], and U-Net [67] have been utilized for their ability to effectively learn from complex medical images to predict metastasis.

Deep learning models, particularly CNNs, have shown increasing advantages in predicting CRC metastasis directly from imaging and histopathology. MRI-based models have been especially effective for LMN prediction in rectal cancer. Wan et al. (2023) demonstrated that a DL model trained on multiparametric MRI significantly outperformed conventional radiological assessment, achieving an AUC of 0.79 for nodal metastasis detection [68].

Liu et al. (2021) [69] used an MRI-based deep learning residual structure to predict distant metastasis among locally advanced rectal cancer patients receiving neoadjuvant chemo-radiotherapy. ResNet18, pre-trained on ImageNet, was fine-tuned using T2-weighted and apparent diffusion coefficient bounding boxes, which were adapted to fit the model's input shape via 1 × 1 convolutions. The models generated probabilities for developing distant metastasis, which were combined into a multi-parametric MR radiomic signature (DLRS) using Cox proportional hazards. DLRS performed well in predicting distant metastasis) [69].

Kindler et al. (2023) [67] designed a deep neural network algorithm for the detection of metastatic CRC using hematoxylin and eosin-stained histologic sections of lymph nodes and evaluated its performance as a diagnostic support tool. They selected 600 whole slide images (WSIs) of benign tissues and 450 WSIs of cancerous tissues, in the form of LMN. The algorithm, developed using a U-Net neural network, achieved an accuracy of 0.952 on slides with cancer and 0.996 on slides with benign samples. The sensitivity of the algorithm was 0.990. This algorithm is suggested to reduce the time needed to predict LMN in CRC specimen without impairing diagnostic accuracy [67].

Lee et al. [15] proposed using CNN generated liver imaging features from abdominal CT images collected from 2019 patients with stage I – III colorectal cancer to predict metachronous liver metastasis. Features were extracted using the top-layer of a pre-trained VGG-16 model. The CNN model, developed based on imaging features and clinical information demonstrated the highest performance (mean AUC = 0.747) for predicting 5-year liver metastasis, compared with a model using clinical features alone (mean AUC = 0.709) [15].

Another study conducted by Wang et al. (2024) [66] developed a preoperative colorectal cancer lymLMN predictive model using deep transfer learning (VGG16), radiomics and clinical features. F18 fluorodeoxyglucose positron emission tomography/computed tomography (18F-FDG PET/CT) scanning was utilized for prediction. Texture features were obtained using methods such as: gray- level co-occurrence matrix (GLCM), gray-level run length matrix (GLRLM), gray level size zone matrix (GLSZM), and neighborhood gray-tone difference matrix (NGTDM). The deep learning radiomics model, integrating deep learning, radiomics and clinical features demonstrated good performance with the area under the curve (AUC) in the training, validation, and test cohorts being 0.934, 0.836, and 0.998 [66].

These findings suggest a potential role for deep learning as a decision-support tool in pathology workflows, reducing diagnostic time without compromising accuracy.

However, not all deep learning applications demonstrated consistent success. Bedrikovetski et al. (2024) [61] developed a deep learning model using a ResNet-50 architecture to predict lymph node status based on standard unenhanced or contrast-enhanced CT preoperatively. A segmentation model was used to predict positive slices in each volume, which were then input into the final classification model for prediction. ResNet-50 achieved an AUROC of 0.619 in the validation cohort and 0.542 in the testing cohort. While the model had higher sensitivity, its specificity was very low. This suggests that ResNet-50 may not be suitable for predicting lymph node status in CRC, potentially due to inter observer variability, varying CT contrast and slice thickness in the training cohort [61]

Zhang, D. et al. (2024) [70] examined a radiomics-boosted deep learning model to evaluate the risk of synchronous peritoneal metastasis (CRPM) in 220 colorectal cancer patients. The objective was to combine radiomic feature maps from PET/CT and image patches through a ResNet50-based deep learning approach to preoperatively predict CRPM. The radiomics-boosted deep learning model achieved high discrimination performance, with AUCs of 0.926 in training, 0.897 in internal validation, 0.885 in external validation, and 0.889 overall, indicating strong potential for identifying patients at elevated synchronous peritoneal metastasis risk.

Xiao, C. et al. (2022) [71] retrospectively included 611 stage I–III colorectal cancer patients, divided into a training cohort of 428 and a validation cohort of 183, to develop a deep learning model using digital hematoxylin–eosin (HE) pathological images for predicting metachronous liver metastasis after surgery. The model showed satisfactory discrimination with C-indexes 0.807 and 0.812 and AUCs 0.840 and 0.848 for predicting 1–3-year probability of liver metastasis in both training and validation cohorts, outperforming conventional clinicopathological models.

Kiehl, L. et al. (2021) [72]used histological whole-slide images from 2431 patients in the DACHS cohort to examine whether deep learning features from primary colorectal tumor histology could predict LMN. An internal test set and external validation set (582 patients from TCGA) were used to evaluate performance. The deep learning slide-based artificial intelligence predictor (SBAIP) achieved an AUROC 0.71 internally and, when combined with clinical data, improved to 0.741, although performance dropped on the external set. These findings suggest deep learning image analysis in LNM prediction with further optimization for external generalizability.

This variability highlights the need for standardized imaging protocols and multicenter validation before clinical application. Table 2 summarizes the difference in performance metrics across imaging modalities and CNN models

**Table 2:** General summary and results of studies using deep learning models for prediction of colorectal cancer metastasis.

Author (year)	Study purpose	Tumor site	Treatment	Imaging modality	CNN model	ghnhmj Number of samples	Results
Liu et al. (2021) [69]	To predict distant metastasis	Rectal cancer	Neoadjuvant chemo-radiotherapy	CT of abdomen and pelvis MRI	ResNet18	Primary cohort: (n = 170) External validation cohort: (n = 65)	DLRS: c-index of = 0.851 (primary cohort), C-index validation cohort= 0.729 Clinical: c-index of = 0.714 (primary cohort), C-index validation cohort= 0.601
Kindler et al. (2023) [67]	To predict lymph node metastasis	Colorectal cancer	Neoadjuvant oncologic treatment who underwent surgery	Histopathology	U-Net	600 WSIs of benign tissues and 450 WSIs of tissues with cancer	Accuracy of 0.952 on slides with cancerous areas Accuracy of 0.996 on slides with benign samples. Sensitivity: 0.990
Lee et al. (2024) [15]	To predict metachronous liver metastasis	Colorectal cancer	Underwent colectomy	CT and clinical information	VGG-16	2019 patients (4096 features were extracted)	CT and clinical information: AUC = 0.747 clinical information alone: AUC = 0.709
Wang et al. (2024) [66]	To predict lymph node metastasis	Colorectal cancer	Had previous radical surgery	18F-FDG PET/CT	VGG19	119 patients (54 with LNMs, 65 without metastases) and a external validation set of 33 patients	Deep learning, radiomics and clinical features had AUC in the training, validation, and test cohorts being 0.934, 0.836, and 0.998.
Author (year)	Study purpose	Tumor site	Treatment	Imaging modality	CNN model	Number of samples	Results
Bedrikovetski et al. (2024) [61]	To predict lymph node status	CRC	Underwent surgical resection for colon cancer	Preoperative CT	ResNet50	Training (n = 401), Validation (n = 100), Testing cohort 1 (n = 500), Testing cohort 2 (n = 200)	AUROC of 0.619 in the validation cohort AUROC of 0.542 in testing cohort.
Zhang et al. (2024) [14]	To predict peritoneal metastasis.	CRC	Surgery	18F-FDG-PET/CT	ResNet34 and ResNet50	Training cohort (n = 123) Internal validation cohort (n = 41) External validation (n = 56)	The AUCs of the radiomics-boosted deep learning model in the training, internal, external validation cohort were 0.926, 0.897, and 0.885.
Wang et al. (2023) [73]	To predict lymph node metastasis	Rectal cancer	-	preoperative MRI	ResNet18, ResNet50, ResNet101, and ResNet152	Training cohort (n = 444) Validation cohort (n = 81) Test cohort (n = 86)	The ResNet101 achieved the best performance in the test set, with an AUC of 0.79
Xiao et al. (2022) [64]	To predict liver metastasis	CRC	Radical colorectal resection	Histopathology	ResNet50	Training cohort (n = 428) Validation cohort (n = 183)	C-indexes of 0.807 and 0.812 and AUCs of 0.840 and 0.848 in the training and validation cohorts.
Kiehl et al. (2021) [74]	To predict lymph node metastasis	CRC	No neoadjuvant chemotherapy	Histopathology and clinical data	ResNet18	Training and validation cohort (n = 1621) Internal test cohort (n = 810) External test (n = 582)	ResNet18 achieved an area under receiver operating characteristic (AUROC) of 71.0%, the clinical classifier achieved an AUROC of 67.0% and a combination of the two classifiers yielded an improvement to 74.1%.

### 5.6 Datasets

In the domains of machine learning (ML) and deep learning (DL), the significance of datasets cannot be overstated, as they serve as the foundational element for training models and achieving optimal performance. Datasets must be large and diverse to ensure that models can generalize well to unseen data. For instance, the ImageNet dataset, which comprises millions of annotated images, is a benchmark for training deep learning models in image classification tasks, demonstrating the necessity of extensive data for effective model training [54], [75]. In medical imaging, while datasets are often smaller, they still require careful annotation and curation; for example, datasets used for lung cancer detection from CT scans are meticulously annotated to enhance model understanding and performance [76]. In this review, most of the studies used private datasets from different hospitals. Few studies used public datasets such as the Surveillance, Epidemiology, and End Results (SEER) database [11], [36], [77] and TCGA [74].

The challenges associated with dataset acquisition, including issues of labeling accuracy and dataset shift, further complicate the landscape of machine learning and deep learning [78].

High-quality datasets enable models to learn intricate patterns and representations, which are crucial for tasks such as image recognition and drug discovery [79]. Techniques like data augmentation can be employed to artificially expand the size of datasets, thereby improving the robustness of models trained on limited data [80]. Additionally, the advent of transfer learning allows models pre-trained on large datasets, such as ImageNet, to be fine-tuned on smaller, domain-specific datasets, thus enhancing performance even when data is scarce. Overall, the interplay between dataset quality, size, and the methodologies employed to leverage them is critical in advancing the capabilities of machine learning and deep learning applications across various fields [81].

#### 5.6.1 Type of data

**Fig. 1:**
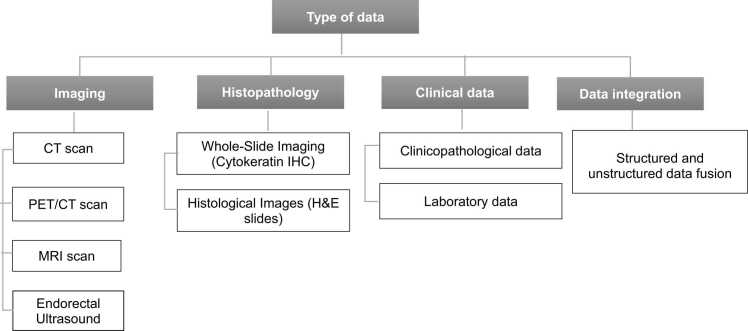
Overview of four main categories of multimodal data types considered.

The predictive modeling of metastasis among CRC patients uses different types of data to enhance accuracy in diagnosis and treatment. Clinicopathological data, including patient demographics, tumor characteristics, and other clinical parameters, provide a foundational understanding of disease progression. Integrated free-text medical records captured unstructured data, such as physician notes and diagnostic results, for comprehensive evaluation of patient history when it is processed through natural language processing techniques. Structured laboratory data such as biochemical and tumor markers, add precision by measuring risk factors. Imaging modalities include contrast-enhanced CT (abdomen and pelvis), MRI scans (T1-weighted, T2-weighted, diffusion-weighted imaging), and 18F-FDG PET/CT, which contribute to detailed metastatic site assessment and metabolic changes. Additionally, histological images from H&E-stained slides contribute cellular-level insights. It is recommended to combine these types of data into fusion models to improve the model’s predictive performance for early detection and personalized treatment strategies [82]. (Fig. 1)

### 5.7 Transfer learning

Transfer learning is a powerful method in deep learning that uses pre-trained models to improve model performance on new but related tasks [83]. A pre-trained model is one that has already been trained with a large amount of data within the same domain as the intended application. Instead of training a neural network from scratch-an approach that requires extensive data and computational resources-transfer learning involves using a model pre-trained on substantially smaller dataset and fine-tuning it for a specific application. Transfer learning is the best option when labeled data are scarce or expensive to obtain as it can speed up convergence and improve network generalization [46].

### 5.8 Comparative analysis of machine learning and deep learning models in predicting colorectal cancer metastasis

Although deep learning methods generally entail higher computational costs compared to traditional machine learning techniques, they often yield superior results in specific contexts. However, there remains a scarcity of comparative studies examining the effectiveness of deep learning versus classical machine learning in predicting colorectal cancer (CRC) metastasis [84].

According to a study conducted by the Harbin Medical University Cancer Hospital utilized eight classical supervised machine learning algorithms, two deep learning algorithms, and one transfer learning model were utilized to predict CRC LMN from a dataset comprising 3364 samples (1646 positive and 1718 negative). The results revealed that deep transfer learning was the most effective method, achieving an accuracy of 0.7583 and an area under the curve (AUC) of 0.7941 [85].

In a recent study by Wang et al. (2024), a prediction model for LMN in CRC patients was developed by integrating clinical data, radiomics features, and deep transfer learning. Feature selection was performed using Least Absolute Shrinkage and Selection Operator (LASSO) regression and the Spearman correlation coefficient. The model incorporated Support Vector Machine (SVM) and Deep Learning Radiomics (DLR). The deep learning-based approach demonstrated superior performance compared to classical machine learning models in predicting LMN [66].

Another study explored the use of radiomics-based classifiers and convolutional neural networks (CNNs) to determine the primary origin of gastrointestinal liver metastases. The radiomics-based K-Nearest Neighbor (KNN) classifier achieved an AUC of 0.87 and an accuracy of 0.67 on an independent test set, outperforming other methods. In contrast, the image-based DenseNet-121 classifier achieved an AUC of 0.80 and an accuracy of 0.83, indicating its strong performance in the classification task [86].

Traditional machine learning techniques, including Naive Bayes, Logistic Regression, Support Vector Machine, Random Forest Classifier, and Multi-Layer Perceptron, have been used for various colorectal cancer metastasis prediction tasks [9], [11], [87], [88]. Deep learning techniques such as CNNs have also been used for data classification, often demonstrating faster processing and improved accuracy compared to manual methods [89], [90].

The comparative analysis of machine learning and deep learning models in predicting colorectal cancer metastasis reveals that while classical machine learning algorithms remain valuable, deep learning models, particularly those utilizing transfer learning and radiomics, tend to offer superior performance. The integration of deep learning with radiomics features such as texture, shape and intensity extracted from medical images, along with clinical data improved predictive accuracy in recent studies. This trend underscores the potential for deep learning approaches to revolutionize the predictive accuracy in cancer metastasis, potentially leading to more effective and timely treatment strategies. However, the computational demands and resource requirements of deep learning models should be carefully considered in clinical settings [91].

## 6 Challenges and solutions

In the context of colorectal cancer metastasis prediction, the availability of quality dataset, interpretability, computational resources and ethical and legal considerations are challenging areas.

### 6.1 Data volume and quality

A large amount of data with high quality is necessary to train millions of parameters in both in DL and ML models. Deep models need a large number of datasets than ML models to avoid overfitting and to develop more generalizable and accurate models [92]. Deep learning models struggle to produce successful results on smaller datasets due to “data hunger”. Several factors contribute to the difficulty of obtaining an adequate number of datasets, including data privacy issues, limited data-sharing agreements, and variability across data sources. Datasets for DL and ML models often encounter limited volume, imbalance and poor-quality data, with issues such as missing values, artifacts, inconsistencies, and noise, which severely affect the performance of predictive models [93]. In this review, the majority of the included studies were conducted based on private datasets from different hospitals and research centers. In contrast, public datasets such as the Surveillance Epidemiology and End Results (SEER) database and the Cancer Genome Atlas (TCGA) cohort were used to collect clinicopathological factors and histological slides respectively. However, there is a lack of public imaging datasets, such as CT, MRI, and PET/CT, specifically for the prediction of colorectal cancer metastasis. A lack of adequate or balanced datasets can lead to models that perform well on training data but fail to generalize to new or unseen data, limiting the clinical applicability of the prediction model. Another significant challenge is availability of large, annotated datasets of CT, MRI, or PET/CT for training the model to early predict colorectal cancer metastasis. These models learn from the annotated colorectal cancer lesion shapes, textures, and intensities. However, annotating large volume of medical data is time consuming and requires skilled professionals such as radiologists. [94]. On contrary, annotating non-medical dataset is simpler and less time taking, as demonstrated with large non-medical datasets like the ImageNet database [54]. The lack of publicly annotated datatsets affect the model prediction capabilities in colorectal cancer metastasis. Therefore, data volume and quality problems should be addressed to improve model prediction performance. Different approaches have been used to overcome the data volume and data quality issues.

• **Image preprocessing:** These methods improve the quality of images by manipulating raw image data to eliminate distortions and enhance specific qualities essential for computer vision applications. Techniques include normalization, resizing (standardizing the image size), noise reduction, contrast adjustment, histogram equalization, and data augmentation [95].
• **Data augmentation:** This approach artificially increases the amount of data by creating slightly changed copies of existing training data without collecting new data [96] [97]. Data augmentation is used to reduce overfitting by increasing the amount of data through augmentation parameters such as rotation, sheering, cropping, zooming, flipping and color-shifting [98]. For example, a study conducted on radiomics-boosted deep-learning to predict the risk of peritoneal metastasis in colorectal cancer patients used data augmentation and transfer learning to address the challenges of training extensive dataset requirement, thereby expanding the dataset and preventing overfitting [99].
• **Transfer learning:** This technique is a powerful tool particularly important when dealing with insufficient data for a new domain, and in the absence of adequate computational resources to build a new model. Annotated data sets are limited in medical imaging particularly for colorectal cancer. Thus, transfer learning is the best option for managing insufficient data by using pretrained model. A pretrained model trained on a large dataset such as ImageNet with over 14 million images, can be adapted to smaller colorectal cancer dataset, thereby reducing computational costs and the complexity of training a new model from scratch. [100], [101]
• **Generative Adversarial Networks (GANs)** can be used to generate artificial data based on the original dataset to solve inadequate or imbalanced colorectal cancer datasets. These artificial data can improve model training, generalization, and reduce overfitting [102]. GANs are also useful in data augmentation, creating diverse and realistic samples that capture the complex variations in metastatic patterns. Therefore, GANs significantly improve the accuracy and reliability of predictive models, contributing to potential advancements in the early detection and treatment of colorectal cancer metastasis [102].

### 6.2 Interpretability and transparency

The interpretability and transparency of predictive models are crucial, especially in clinical applications of AI where understanding the reason behind a decision is as important as the decision itself. Interpretability and transparency ensure impartiality in decision-making by detecting and correcting biases in the training dataset. They increase the robustness of models by identifying potential issues that could affect predictions, allowing for adjustments that improve model reliability. Furthermore, they serve as safeguards to ensure that the model's outcome is based on meaningful and relevant variables. This helps to build trust in the model's predictions and supports clinical applications where interpretability and transparency are essential for critical clinical decision-making. [103] Deep learning models, despite their effective predictive capabilities, the effect of millions of parameters on the result makes it a "black box" providing little information about how specific features contribute to the output. It is vital for a model to provide explanations for a given output. Medical experts need detailed information about the predictive model to support the proposed diagnosis or prediction rather than a simple binary prediction. In contrast, several machine learning models, such as decision trees or linear models, provide better interpretability and transparency [100].

Thus, several solutions have been proposed to address these challenges. For example, one effective approach is the use of explainable AI (XAI) which provides insights into model decision-making processes. Methods such as SHAP (SHapley Additive exPlanations) and LIME (Local Interpretable Model-agnostic Explanations) can explain how individual features affect a model’s predictions. These methods have been shown to improve the interpretability of complex models, by providing detailed and understandable explanations of model predictions for healthcare professionals. In addition, incorporating clinical insights and biological understanding into the feature selection process, models can be designed to focus on clinically relevant variables, thereby enhancing their transparency and interoperability. This approach ensures that the model's outcome aligns with established medical knowledge [104].

Moreover, the development of hybrid models that combine interpretable ML algorithms such as decision trees or linear regression with deep neural networks can improve predictive capabilities and interpretability. For example, using ensemble methods that combine interpretable and non-interpretable models can provide the best predictions while maintaining transparency [105].

### 6.3 Computational resources

The training and deployment of ML and DL models, especially those that use complex architectures such as convolutional neural networks (CNNs), requires substantial and often expensive computational resources, including high-performance GPUs, large memory, and extended processing times. These requirements lead to AI models accessibility challenges, particularly in research environments with limited resources or in low- and middle-income countries where such infrastructure is not readily available in all clinical settings [106]. On the contrary, several traditional ML models, while still demanding resources, generally require less computational power and are more feasible to implement in various settings. The difference in resource requirements between ML and DL models indicates the need for careful consideration of the feasibility and accessibility of these technologies in different research and clinical environments.

It is necessary to optimize computational resources to overcome the challenges associated with computational demands, researchers can use techniques such as transfer learning, which allows models pre-trained on large datasets to be fine-tuned on smaller datasets. In addition, cloud computing services can be used to obtain scalable resources without the need for extensive hardware infrastructure. Furthermore, developing efficient algorithm design, such as the use of lightweight models or composite networks that utilize unsupervised learning, can reduce the computational burden. These models can extract features from large volumes of non-annotated data, thereby reducing the need for large, annotated datasets. The majority of the studies included in this review did not use high-performance GPUs due to limited datasets that did not require high computational resources; however, they have utilized computers with large memory processing to predict colorectal cancer metastasis [107].

### 6.4 Ethical and legal considerations

The use of imaging and clinical data in DL and ML models raises concerns about patient privacy, data security, and potential algorithmic biases. Addressing these concerns is necessary to ensure adherence to ethical principles. For example, if an AI model is trained on a dataset that lacks diversity, it cannot be generalized to different populations leading to inequitable health outcomes [108], [109]. Moreover, the use of AI prediction models for clinical decision-making processes is not fully accountable for errors, which may violate the legal standards designed to protect patient rights [110] Early prediction of colorectal cancer metastasis involves a complex process of data annotation and algorithm development in which even a minor error can affect predictions. Clinicians would like to use AI models to early predict colorectal cancer metastasis, but the “black box” nature of these models complicates its understandability and trust which may potentially affect patient’s autonomy and consent [111]. Therefore, regulatory frameworks that govern the use of AI in health care are essential to ensure accountability and responsibility while deploying AI in healthcare systems. This includes promoting transparency in AI decision-making processes using explainable AI (XAI) techniques, which clarify how ML and DL models make predictions [108], [112]. Additionally, it is important to involve stakeholders such as clinicians, patients, and ethicists in the development and evaluation of AI systems to address biases and ethical issues early in the process [109]. Policymakers should be engaged to create standards that ensure the ethical use of AI systems, build trust, and facilitate the integration of AI systems into clinical practice [108], [109].

## 7 Outlook

Recent developments in model architectures including GANs transformer models, have demonstrated their effectiveness in improving predictive performance. For example, GANs can be used in the future to generate artificial medical images, which can increase the size and variability of datasets, thereby improving model performance and generalizability [113]. Additionally, transformer-based domain-specific foundation models like OncoBERT can be used for survival prediction in cancer patients, indicating the efficacy of pre-trained models in transfer learning [113].

Recent studies have demonstrated the efficacy of different ML algorithms, such as random forest and Light Gradient Boosting in predicting the risk of liver metastasis in rectal cancer patients [36]. Similarly, Liang et al. found that logistic regression achieved higher predictive performance than support vector machines in the prediction of metachronous liver metastases among rectal cancer patients. This indicates that future studies should use hybrid models combining traditional statistical methods alongside advanced machine learning techniques to enhance predictive accuracy [114].

The integration of AI into clinical practice is supported by the development of deep learning models, which are capable of processing multimodal data, such as combining imaging, clinicopathological, genomic, and epigenomic data. This approach provides a more comprehensive understanding of tumor biology and patient-specific factors, which is necessary for predicting metastasis and achieving high performance metrics [115]. For example a DL model (Deep2Met) developed using DNA methylation to predict CRC metastasis demonstrated a promising result, with an area under precision-recall curve (AUPR) and an average F-score of 96.99% and 94.71%, respectively [116]. Additionally, the use of gene expression data in machine learning algorithms is important for identifying the main biomarkers that could serve as a predictor of metastasis among CRC patients [117]. This indicates that future studies should focus on incorporating molecular data into the model to improve predictive model performance. Moreover, the application of transfer learning and domain adaptation methods helps to utilize models trained on larger datasets to be fine-tuned on smaller dataset for specific clinical use in the future [118]. A multicenter prospective study should be conducted in the future to validate the model’s performance in larger and diverse patient populations and to increase generalizability.

The impact of these emerging technologies on patient outcomes is profound. The quality of life and survival rate of colorectal cancer patients will be enhanced by improving the accuracy of metastasis prediction models. Studies have revealed that AI-based risk predictive models performed better than traditional methods, thereby facilitating timely and appropriate interventions [119]. Furthermore, the ability to generate personalized treatment plans based on predictive analytics can lead to more effective therapeutic strategies, ultimately improving patient outcomes [120].

In addition to clinical benefits, the integration of AI in cancer care has the potential to reduce healthcare expenses. AI can reduce unnecessary procedures and hospitalizations by supporting accurate treatment planning, thereby alleviating the financial burden on healthcare systems [121]. Thus, the use of AI technologies to predict metastasis among colorectal cancer patients not only improves individual patient outcome but also contributes to a more sustainable healthcare model.

## 8 Conclusion

This comprehensive review included 38 articles to evaluate current advancements in ML and DL models for predicting metastasis among colorectal cancer patients. These models have shown a great potential in predicting common metastatic sites, such as lymph nodes and the liver, as well as less frequent sites like the lungs, bones, and peritoneum.

The reviewed models included traditional ML algorithms such as logistic regression, support vector machines, and random forests, as well as advanced DL architectures such as CNNs, including ResNet, VGGNet, and U-Net, which have been found effective in learning complex data patterns from different clinical, imaging, and histological data. The combination of multimodal data, such as imaging and clinical data, and the application of transfer learning have further enhanced the performance of these predictive models, supporting early and individualized treatment strategies that can improve patient outcomes. However, challenges such as the scarcity of high-quality and large-scale datasets, computational resource, limitations transparency and interpretability of complex models, and ethical considerations about data use have affected the widespread clinical application of these models.

The comparative analysis highlighted the superior performance of DL models over traditional ML models, especially when combined with radiomics and clinical features. However, the increased computational costs and resource requirements of DL techniques need careful consideration in clinical applications. Unlike earlier studies mainly focused on the detection or staging of metastasis, this review purposively identified performance variations due to specific metastatic characteristics across organ sites.

More recent studies favor radiomics-driven multimodal frameworks, aligning with trends reported in recent large-scale analyses, whereas our review specifically addressed their inconsistent strategies of validation and limited clinical application. This review has several limitations, including heterogeneity in datasets, evaluation metrics, and study design, which have restricted the feasibility of quantitative meta-analysis.

Future research should focus on addressing these challenges through the development of explainable AI models, optimization of computational resources, and establishment of ethical frameworks. The review highlighted the importance of increasing AI models accuracy, transparency and interpretability, allowing health care providers to understand decision making processes of the algorithms. Additionally, it is recommended to validate AI models performance on a multi-center, larger and more diverse patient populations, while exploring the integration of emerging technologies such as GANs and transformer models, as well as the inclusion of molecular, genomic and epigenomic data, to further refine predictions and, improve the accuracy and clinical application of predictive models.

## CRediT authorship contribution statement

**Mikiyas Amare Getu:** Writing – original draft, Visualization, Validation, Methodology, Conceptualization. **Tesfaye Amare:** Methodology, Data curation. **Kefeng Li:** Writing – review & editing. **Anam Mehmood:** Writing – review & editing, Visualization. **Yonas Fissha Adem:** Writing – review & editing, Visualization. **Pablo Santos:** Writing – review & editing. **Hu Yan:** Writing – review & editing, Supervision.

## Human and animal rights

This article does not contain any studies involving animals performed by any of the authors.

## Consent for publication

Not applicable.

## Informed consent

This article does not contain patient data.

## Ethical approval

This article does not contain any studies with animals performed by any of the authors.

## Funding

This research was supported, in part, by the 10.13039/501100010295NORA Consortium (Network for Oncology Research in Africa, https://www.umh.de/nora), funded by the German Federal Ministry of Research, Technology and Space (grant 01KA2220). This research was supported, in part, by the REACCT-CAN Consortium, funded by the Science for Africa Foundation to the Developing Excellence in Leadership, Training and Science in Africa (DELTAS Africa, program De-22–008), with support from the Welcome Trust and the UK Foreign, Commonwealth and Development Office. This research was supported, in part, by the EDCPT2 program, supported by the European Union and the Global and; Planetary Health Working Group of the Martin Luther University Halle-Wittenberg (Grant number 81295996).

## Declaration of Competing Interest

The authors have no conflict of interest to declare.

## Data Availability

The datasets used and/or analysed during the current study are available from the corresponding author on reasonable request.
